# The research progress and research trends in acute coronary syndrome nursing: A review of visual analysis based on the Web of Science database

**DOI:** 10.1097/MD.0000000000035849

**Published:** 2024-02-16

**Authors:** Jialong Liu, Chaojun Li, Wanping Mei, Hanzhi Qin

**Affiliations:** a School of Nursing, Anhui University of Chinese Medicine, Hefei, China; b Department of Nursing, the First Affiliated Hospital of University of Science and Technology of China, Hefei, China.

**Keywords:** acute coronary syndrome, nursing, research frontiers, research hotspots, visual analysis

## Abstract

Acute coronary syndrome (ACS) is one of the most common and severe forms of cardiovascular disease and has attracted worldwide attention with increased morbidity and mortality in recent years. There are few review studies in the field of its care in the form of bibliometric studies. We searched the Web of Science Core Collection database for articles and reviews in the area of ACS nursing for visual mapping analysis. Our objectives are to explore the hot topics and frontiers of research in the field of ACS nursing and to identify collaborative relationships between countries, institutions, and authors. This study will provide researchers with intuitive reference data for future in-depth studies of ACSs.

## 1. Introduction

Acute coronary syndrome (ACS) is an acute ischemic syndrome of the heart caused by thrombosis due to rupture or erosion of unstable atheromatous plaques in the coronary arteries.^[1–3]^ It is more common in western countries such as Europe and the United States, with a higher incidence in men than in women, and is also a major cause of death in people.^[3,4]^ Approximately 19 million people died from cardiovascular disease worldwide in 2020, an increase of 18.7% compared to 2010.^[5]^ ACS is one of the most common and serious conditions of cardiovascular disease, which has attracted increasing attention worldwide in recent years. The care of ACS plays an important role in reducing readmissions and mortality. Studies have shown that the implementation of ACS care pathways can help minimize treatment delays and improve patient prognosis without increasing the additional financial burden on patients.^[6]^ At present, the care of ACS patients has received more attention from scholars, and the relevant review literature is mainly from the diagnostic and treatment measures, risk prevention, disease management and other aspects of the exposition and summary,^[7–10]^ and very few studies have used the form of visual mapping analysis to intuitively reflect the hotspot of the research and research frontiers of the care of ACS. CiteSpace is the development of visual analysis software in the context of scientometrics and data visualization, which allows authors in a particular field of research to analyze their data. It can qualitatively and quantitatively analyze the authors, institutions, countries, journals, and co-cited literature of a certain research field, and intuitively reflect the research hotspots and research frontiers of a certain research field in the form of dynamic mapping.^[11–13]^ This advantage cannot be realized by traditional reviews. Therefore, this study intends to use bibliometric research methods to demonstrate the research hotspots and development trends in the field of ACS nursing in the past 10 years in the form of visual mapping by visualizing countries, institutions, authors, co-cited literature, and keywords, so as to provide a reference for the future research on ACSs.

## 2. Materials and methods

### 2.1. Data source

**Figure 1.**
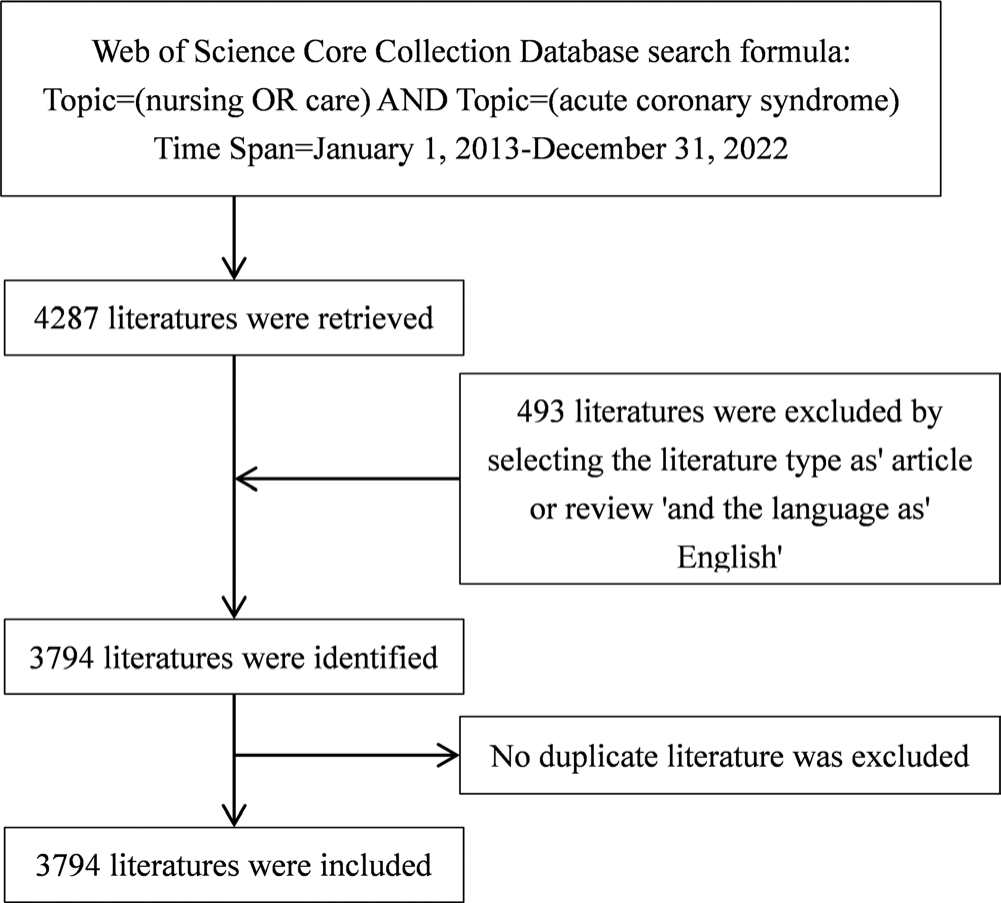
Flow chart of literature screening included in this study.

The Web of Science Core Collection database was searched with the search terms “acute coronary syndrome,” “nursing,” “care.” The time period was from January 1, 2013 to December 31, 2022. The initial search yielded 4287 articles, and 3794 articles were included in the database by targeting the type of article or review and the language “English.” The selected literature was downloaded on May 13, 2023, (see Supplemental Digital Content, http://links.lww.com/MD/L566, http://links.lww.com/MD/L568, http://links.lww.com/MD/L570, http://links.lww.com/MD/L572, http://links.lww.com/MD/L574, http://links.lww.com/MD/L575, http://links.lww.com/MD/L576, http://links.lww.com/MD/L577). The literature screening process is shown in Figure 1.

### 2.2. Data analysis and processing methods

The included literature was visualized and analyzed using Citespace 6.2.R2, a software for bibliometric studies developed by the team of Professor Chao-Mei Chen at Drexel University.^[14]^ No duplicate literature was shown after it was checked. The time slices were set to 1 year, and the top 100 keywords were selected for each time slice for visual analysis, and the cluster labels were extracted using the “LLR (Log-Likelihood Ratio) algorithm.” For the analysis of highly cited literature, Top 50 was selected for each time slice, and k = 20 was selected for the visualization of authors, institutions, and countries, and “Minimum Spanning Tree and Pruning sliced networks” was used for cropping. The ethical approval was not applied in current study because there was no patient privacy or clinical samples.

## 3. Results

### 3.1. Distribution of the number of articles issued

**Figure 2.**
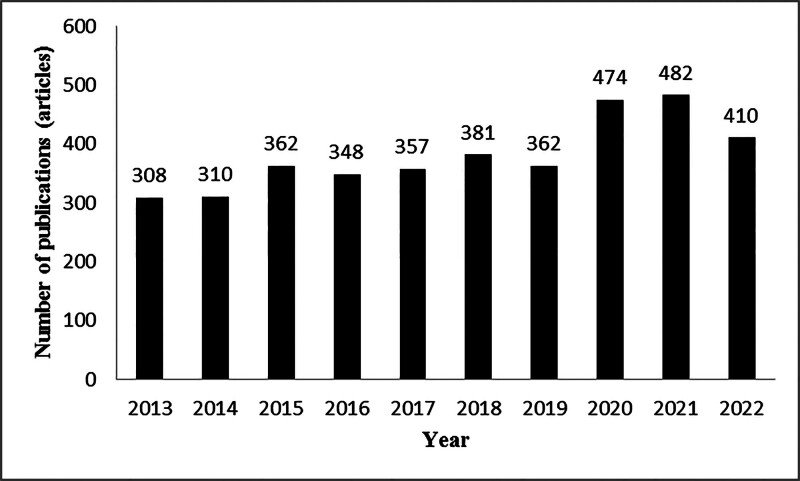
The annual number of publications in the field of acute coronary syndrome nursing.

The annual publication volume of ACS nursing research in the Web of Science database from 2013 to 2022 is shown in Figure 2. The overall trend of publication volume in this field is steadily increasing, with a surge in publication volume manifested in 2020 to 2021.

### 3.2. Visual mapping of countries and institutions

**Figure 3.**
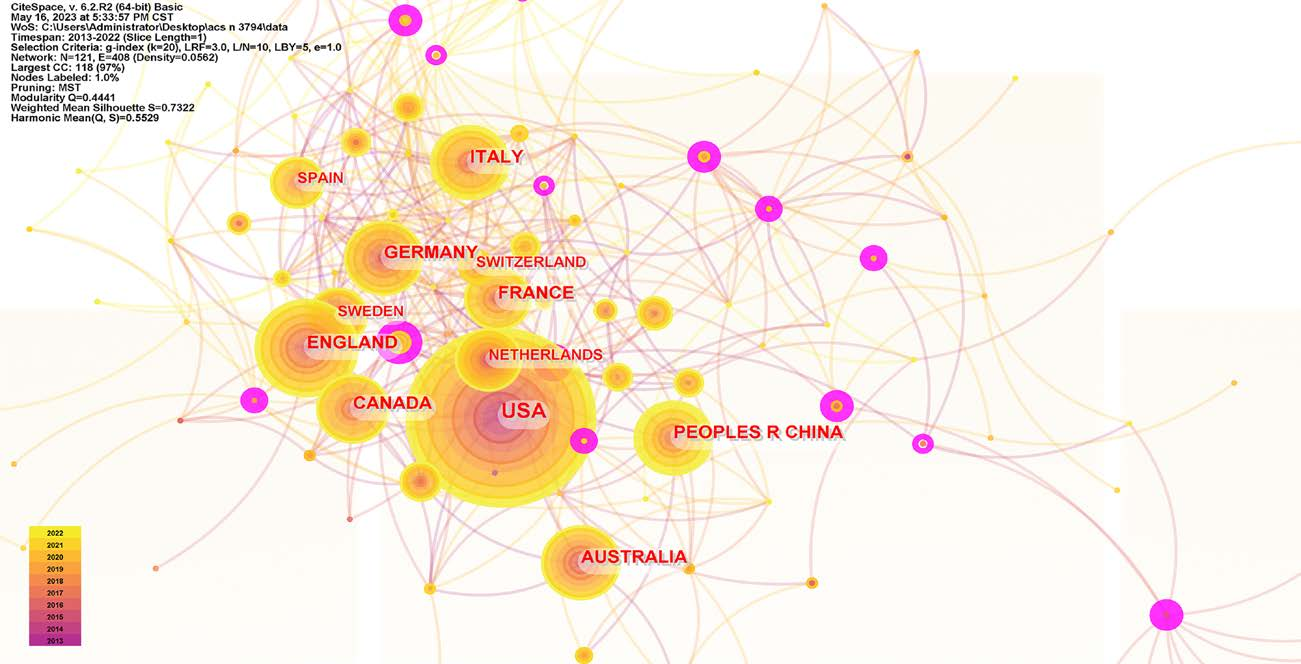
Country visualization mapping.

**Figure 4.**
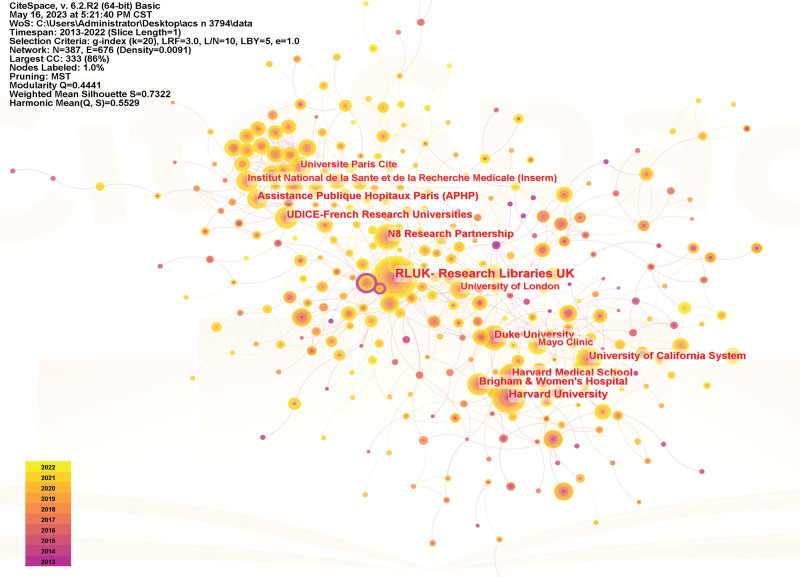
Institution visualization mapping.

The circle in the figure indicates each country or institution node, the larger the area, the more publications; the connecting line indicates a cooperative relationship; when the intermediary centrality of the node is > 0.1, the circle will appear purple outer circle, which represents that the node has an important role in the process of information transmission.^[12–14]^ The results show that a total of 121 countries and 387 institutions have published literature on ACS care research, and there is strong communication and cooperation between institutions in each country. The top 5 countries in terms of the number of publications USA (1319), England (436), Australia (326), Italy (311), Canada (309), and China (279) ranked 7th; the top 5 countries in terms of centrality Togo (0.25), South Africa (0.21), Mexico (0.18), Tunisia (0.16) and Senegal (0.15). The top 5 institutions in terms of publications RLUK-Research Libraries UK (403), Harvard University (236), Harvard Medical School (173), N8 Research Partnership (157), Duke University (156 articles); top 3 institutions in terms of centrality Uppsala University (0.27), University of Basel (0.12), Hebrew University of Jerusalem (0.11). The countries and institutions visualization maps are shown in Figures 3 and 4.

### 3.3. Visual mapping of authors

**Figure 5.**
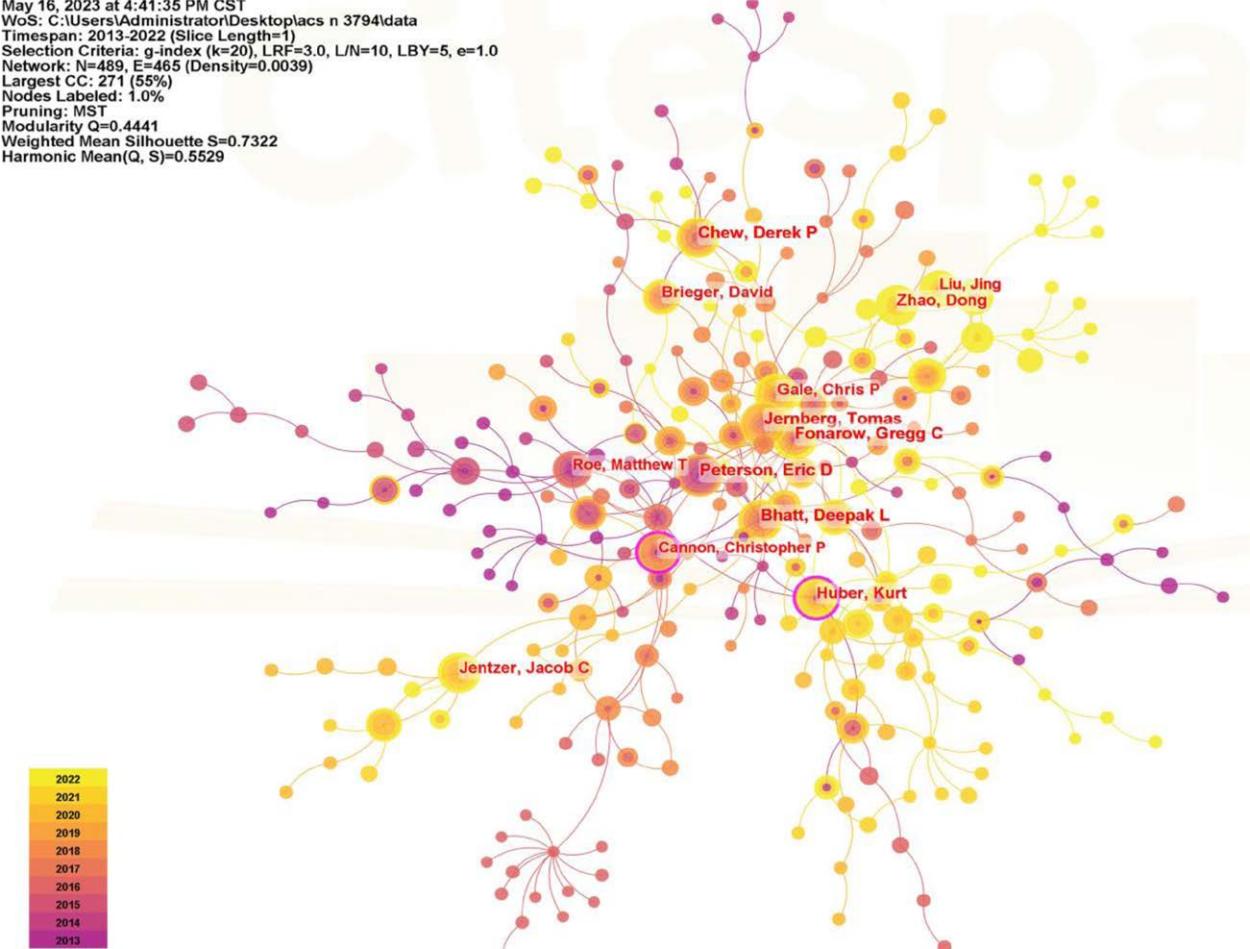
Author visualization mapping.

The communication among core authors and their scientific ability have an important impact on the quality of articles. According to Price law, the core authors of the published articles are judged by M ≈ 0.749(Nmax)1/2, M is the minimum number of published documents of the core authors, and Nmax is the maximum number of published documents of the authors. After calculation, the number of published articles is not <14 as the core authors in this field, totaling 29. Among them, the top 5 authors in terms of number of publications are Fonarow, Gregg C (37), Chew, Derek P (37), Jernberg, Tomas (37), Peterson, Eric D (36), Bhatt, Deepak L (35). A collaborative team centered on Huber, Kurt (30 articles), Cannon, Christopher P (23 articles) was formed. The author visualization map is shown in Figure 5.

### 3.4. Keyword analysis

#### 3.4.1. High-frequency keywords.

The keywords in the field of ACS care were visualized and analyzed, and 265 nodes and 885 links were obtained. The top 5 keywords in terms of frequency: “acute coronary syndrome” (1419 times), “acute myocardial infarction” (929 times), “myocardial infarction” (826), “management” (621), and “outcome” (590); and the top 5 keywords in terms of centrality: “diagnosis” (0.15), “acute coronary syndrome” (0.12), and “percutaneous coronary intervention” (0.12), “management” (0.1), and “validation” (0.09). By analyzing the high-frequency keywords, it can be seen that the current research on ACS care mainly focuses on patient outcome management, early predictive identification and diagnosis and validation studies, and postoperative care after percutaneous coronary intervention.

#### 3.4.2. Cluster analysis of keywords.

**Figure 6.**
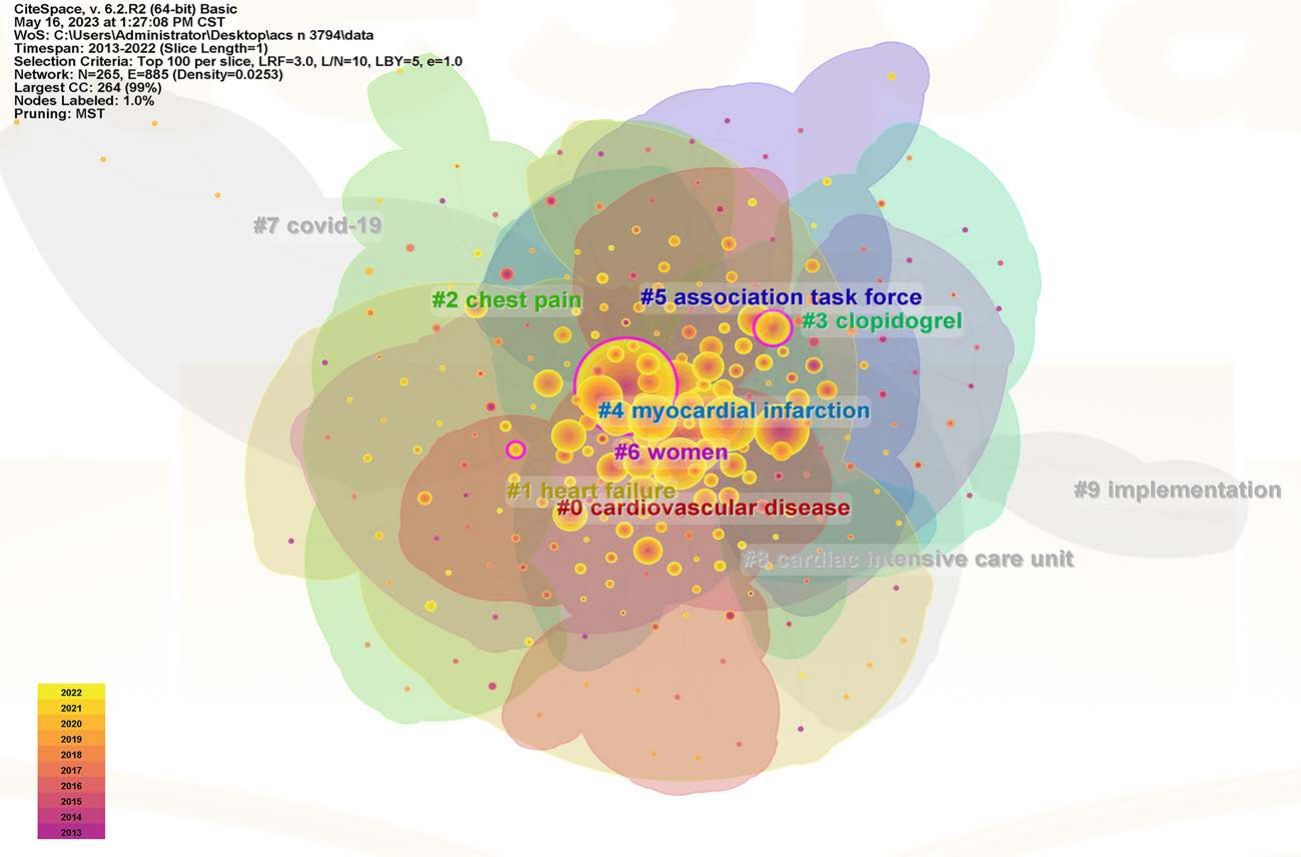
The network of keyword clustering.

**Figure 7.**
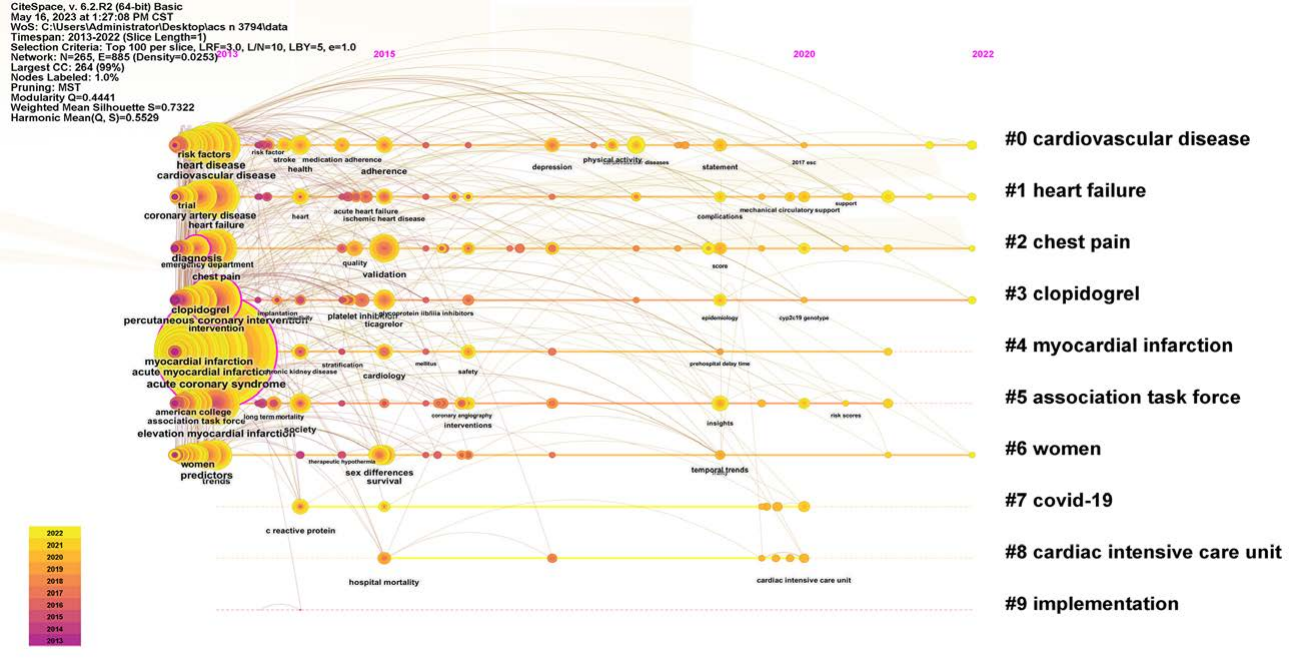
Timeline chart of keywords.

Cluster analysis of keywords can reflect the research hotspots in the field to a certain extent.^[15]^ The timeline graph can show the initial appearance time and duration of each keyword, and the closer the color is to the warmer shade of yellow, it means that the content is still in the research stage in recent years. The results show 10 clusters, including: #0 cardiovascular disease, #1 heart failure, #2 chest pain, #3 clopidogrel, #4 myocardial infarction, #5 association task force, #6 women, #7 covid-19, #8 cardiac intensive care unit, #9 implementation. The Modularity Q and the Mean silhouette S of the results are the reference metrics to judge the effectiveness of the mapping. In this study, Q = 0.4441 > 0.3 and S = 0.7322 > 0.7, indicating good homogeneity and representing the research hotspot in the field of ACS care. The keyword clustering mapping and timeline diagrams are shown in Figures 6 and 7.

#### 3.4.3. Bursting keywords.

**Figure 8.**
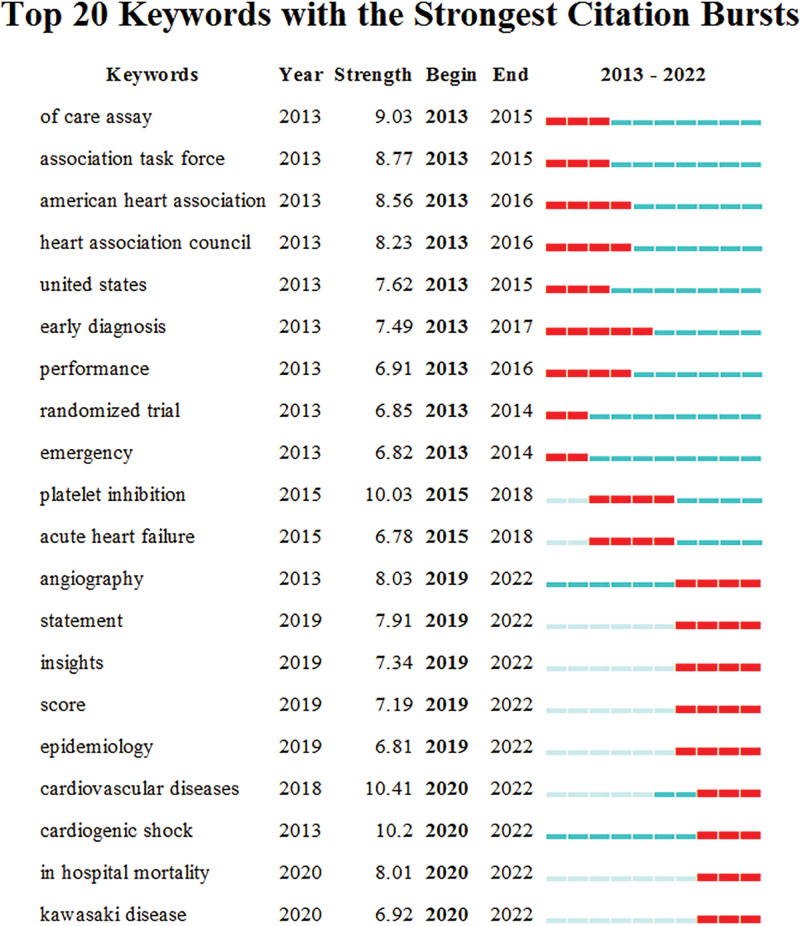
Top 20 keywords with the strongest citation bursts.

The burst words refer to the words with significantly higher frequency of use or sudden increase in a short period of time, which can reflect the research frontier and development trend of a certain research field to a certain extent. The red line in the figure indicates the time period of keyword bursts.^[13]^ The top 20 burst words with higher weights in the field of ACS care from 2013 to 2022 are shown in Figure 8. The research hotspots from 2013 to 2015 are mainly focused on immediate measurement of Cardiac Troponin, heart association, United States, early diagnosis, performance, randomized trial, emergency; and from 2016 to 2018, the research focus was mainly on platelet inhibition and acute heart failure; 2019 to 2022, angiography, statement and insights, risk scores, epidemiology, cardiovascular disease, cardiogenic shock, hospital mortality, and Kawasaki disease have dominated ACS care research in recent years.

### 3.5. Highly cited literature

Highly cited literature is highly recognized literature in the same field of study, and exploring its patterns can reflect the research frontiers in the field to some extent. We analyzed the cited literature and listed the top 5 highly cited literature in the field of ACS care by citation frequency, which were, in order, Roffi M (2016)^[16]^ 329 times, Ibanez B (2018)^[17]^ 243 times, Hamm CW (2011)^[18]^ 185 times, Steg PG (2012)^[19]^ 181 times, Amsterdam EA (2014)^[20]^ 152 times, as detailed in Table 1. The most cited literature,^[16]^ published in 2016, was a guideline update by Roffi M of all the available evidence from the 2011 European Society of Cardiology on the management of ACSs in patients without persistent ST-segment elevation. This guideline focuses on the management of ACSs in patients without persistent ST-segment elevation in terms of diagnosis, risk assessment and outcomes, treatment, and summary of management strategy. The second highly cited literature was published in 2018 by Ibanez B.^[17]^ This guideline is an update of the 2012 European Society of Cardiology guideline on the management of acute myocardial infarction in patients with ST-segment elevation, addressing aspects such as emergency care, reperfusion therapy, management during hospitalization and at discharge, long-term therapies for ST-segment elevation myocardial infarction, complications following ST segment elevation myocardial infarction, myocardial infarction with non-obstructive coronary arteries, and assessment of quality of care are important in guiding the management of acute myocardial infarction in patients with ST-segment elevation. The development of each guideline lays the foundation for high-quality cardiovascular care while providing recommendations for selecting the optimal management strategy for a given patient. Meanwhile, the top 5 highly cited literature shows that articles in the European Heart Journal have the highest number of citations and the highest impact factor, indicating that the guidelines for the management of patients without persistent ST-segment elevation ACS and ST-segment elevation acute myocardial infarction published by the European Heart Association have important academic reference value in the field of ACS care.

**Table 1:** Highly cited literature in the field of acute coronary syndrome nursing.

Rank	Citation frequency	Yr	Title	Journal	IF (2022)
1	329	2016	2015 ESC guidelines for the management of acute coronary syndromes in patients presenting without persistent ST-segment elevation	European Heart Journal	39.3
2	243	2018	2017 ESC Guidelines for the management of acute myocardial infarction in patients presenting with ST-segment elevation	European Heart Journal	39.3
3	185	2011	ESC Guidelines for the management of acute coronary syndromes in patients presenting without persistent ST-segment elevation	European Heart Journal	39.3
4	181	2012	ESC Guidelines for the management of acute myocardial infarction in patients presenting with ST-segment elevation	European Heart Journal	39.3
5	152	2014	2014 ACC/AHA guideline for the management of patients with non-ST-elevation acute coronary syndromes	Journal of the American College of Cardiology	24

ACC = American College of Cardiology, AHA = American Heart Association.

## 4. Discussion

### 4.1. Current situation in the field of ACS nursing research

The study showed an overall steady upward trend in the number of publications in the field of ACS care from 2013 to 2022, with a sudden increase in the number of publications in this field, especially in 2020 and 2021, which, combined with the appearance of COVID-19 in the keyword clustering, suggests that the increase in the morbidity and mortality of ACS during the new coronary epidemic has caused the researchers’ attention to the care of ACS to be also elevated.^[21,22]^ In the analysis of country, institution, and author visualization, the attention of ACS care research is related to the economy and medical level of the country where it is located, and the countries with developed economy and higher medical level are more enthusiastic about ACS care research. ACS care research in developed countries in Europe and the United States began relatively early and more mature, as early as 2000, the American College of Cardiology and the American Heart Association for unstable angina and non-ST-segment elevation myocardial infarction patients to formulate management guidelines.^[23]^ As seen in the centrality ranking, developed countries have relatively few links with other countries. On the contrary, less developed countries have more communication links with other countries and turn out to be centers of information transfer, playing an important role in the transfer of information between countries or institutions. Three of the 5 institutions with the highest number of publications were universities, as were the top 3 institutions in terms of centrality, suggesting that research related to ACS nursing is concentrated in universities. RLUK- Research Libraries UK, Harvard University, Harvard Medical School, N8 Research Partnership, and Duke University published the most articles and worked closely with each other to make a significant contribution to the development of the field of ACS nursing. The close cooperation between countries, institutions, and authors is conducive to the rapid development of the field of ACS nursing.

### 4.2. Analysis of research hotspots in the field of ACS nursing

A high-frequency and cluster analysis of keywords yielded that the research hotspots in the field of ACS nursing are mainly focused on the early predictive identification and diagnosis of the disease, postoperative care after percutaneous coronary intervention, pharmacological treatment, and patient management. Early recognition is essential for the optimal management of ACS, and chest pain is an early symptom of ACS whose recognition and early intervention are critical in nursing.^[24]^ For the early identification and diagnosis of patients with ACS, an increasing number of studies have focused on the use of biomarkers to make judgments, among which immediate measurement of cardiac troponin is a common method.^[25,26]^ Some studies have shown that the heart fatty acid binding protein also has potential diagnostic value in the early period after the onset of ACS symptoms,^[27,28]^ but further validation studies on new predictors are needed to identify and diagnose patients as early as possible, and to take early interventions to improve therapeutic outcomes and survival rates. Percutaneous coronary intervention, as a special treatment modality, significantly solves the reperfusion situation of ACS patients, and its post-procedure care and recovery have also attracted much attention. Most studies have focused on anticoagulation therapy after recanalization of occluded vessels, postoperative use of antiplatelet agents, and prevention and management of postoperative complications.^[29–32]^ These aspects of research are closely related to the improvement of disease status and quality of life in ACS patients after percutaneous coronary intervention. In addition, antiplatelet agents are one of the important therapeutic measures for ACSs, and dual antiplatelet therapy reduces the incidence of cardiovascular events in patients with ACSs.^[33]^ A randomized trial demonstrated that short-term 6-month dual antiplatelet therapy is safe for ACS patients undergoing percutaneous coronary intervention.^[34]^ It is worth noting that clopidogrel, one of the commonly used drugs for treatment, needs further research on how and what to use, despite its superiority to antiplatelet agents such as aspirin and ticagrelor in preventing future adverse events.^[35,36]^ Therefore, how to better use clopidogrel to improve the therapeutic effect and reduce adverse events has become a hot research topic today.

In addition, female ACS patients have some special conditions compared with male patients, including differences in clinical manifestations, pathophysiologic conditions and prognosis of disease course, which make the diagnosis and treatment of female ACS more challenging than that of male.^[4,37,38]^ In recent years, how to carry out rational patient management and improve the therapeutic effect of ACS has also become the key to research. Various guideline studies have conducted in-depth analyses, research and explorations on the diagnosis, treatment, symptom relief, functional recovery, optimization of drug therapy, quality of life and prevention of adverse events for patients with ACS in order to improve patient survival and quality of life.^[7,39,40]^ Meanwhile, patients with ACS are frequently complicated with heart failure, which leads to more severe physical symptoms, and it is closely associated with higher mortality and more frequent readmission of patients.^[41]^ Therefore, there is an urgent need for a large number of studies to explore the management strategies for such patients in order to effectively improve the heart failure symptoms and clinical outcomes of ACS patients with concomitant heart failure, so as to improve the survival rate of patients. In addition to this, nursing management and prognostic management, as one of the focuses of development in the field of ACS care, still need to be studied in depth in the future to improve the effectiveness and quality of clinical care.

### 4.3. Research trends in the field of ACS nursing

With the continuous development of interventional techniques, angiography has been more and more widely used in the diagnosis and treatment of ACSs, which is important in guiding patient treatment and assessing patient prognosis.^[42,43]^ Notably, studies on coronary computed tomography angiography have shown that although it can significantly shorten the diagnostic time in patients with suspected ACS, it may also lead to an increase in invasive procedures to some extent.^[44]^ It has also been suggested that early computed tomography angiography within approximately 6 hours does not change 1-year clinical outcomes in intermediate-risk patients with acute chest pain and suspected ACS, but rather increases the length of hospitalization.^[45]^ Therefore, it has become particularly important to seek a safer and more rapid and sensitive diagnostic strategy to minimize harm to patients. In addition, a growing body of research and practical experience is continually refining healthcare professionals’ understanding and treatment of ACS. Research guidelines and statements issued by relevant organizations play an important role in guiding the clinical treatment and care of patients with ACS, and their constant updating reflects the hot research trends in the field.

In recent years, with the continuous development of research methods and techniques, epidemiologic studies and studies on the construction of risk scoring systems have been increasing in the field year by year. Studies have shown that continuously improved and refined risk scoring systems help to better assist in early diagnosis and guide clinical care practices,^[46,47]^ and epidemiologic studies have provided strong support for the development of more scientific prevention and treatment strategies.^[48,49]^ Today, despite substantial progress in the diagnosis and treatment of ACS, cardiovascular disease remains the leading cause of death worldwide.^[7]^ Among them, ACS complicating cardiogenic shock is a problem that requires special attention during the treatment of ACS patients. Although existing studies have made efforts in early hemodialysis and temporary mechanical circulatory support, the mortality rate of patients with ACS complicating cardiogenic shock is still very high.^[50]^ Some studies have summarized the management of cardiogenic shock complicated by ACS, which mainly includes early shock recognition, optimal selection and initiation of mechanical circulatory support, early coronary revascularization, shock team and implementation of standardized protocols, which play an important role in improving the outcome of cardiogenic shock in patients with ACS.^[51,52]^ However, clinical practices for the management of cardiogenic shock are still inconsistent and knowledge gaps remain large, and it has become a future research trend to conduct systematic studies to explore the selection and timing of mechanical circulatory support and to standardize the medical care and management of patients with ACS complicated by cardiogenic shock.

Kawasaki disease, an inflammatory disease of young children, is the most common cause of acquired heart disease in children in the developed world, and it can lead to serious consequences such as structural abnormalities of the heart and coronary vasculitis.^[53–55]^ In recent years, the incidence of Kawasaki disease has risen to varying degrees, and an increasing number of patients with childhood-onset Kawasaki disease are transitioning to adulthood. Currently, the limited experience of healthcare professionals in recognizing Kawasaki disease in adults poses a serious challenge to medical treatment and care.^[55]^ How to better diagnose and treat Kawasaki disease and manage its vascular complications has become an important future research direction in the field of ACS nursing.

### 4.4. Limitations

There are some limitations in this study; we only searched and downloaded literature from the Web of Science Core Collection database for analysis, which makes our data may not be representative of all literature to some extent. At the same time, only English literature was included in the screening process, which may have made our analysis incomplete. Therefore, more database literature should be included in future studies to further analyze research advances in the field of ACS nursing.

## 5. Conclusion

This study used Citespace software to visualize and analyze the field of ACS care research in the last 10 years based on the Web of Science database. Research in this field is mainly concentrated in developed countries and universities, with strong communication and cooperation between countries and institutions. Nowadays, the research trends in this field are mainly focused on angiography, construction of risk scoring system, management of cardiogenic shock, and adult Kawasaki disease. Through the literature visualization and analysis, we clearly show the current research status in the field of ACS care and analyze the research frontiers and development trends in this field to provide a broad perspective for the research related to ACS.

## Author contributions

**Conceptualization:** Jialong Liu, Hanzhi Qin.

**Data curation:** Jialong Liu, Hanzhi Qin.

**Formal analysis:** Jialong Liu, Chaojun Li, Wanping Mei.

**Methodology:** Jialong Liu, Chaojun Li, Wanping Mei.

**Software:** Jialong Liu, Chaojun Li.

**Supervision:** Hanzhi Qin.

**Validation:** Wanping Mei, Hanzhi Qin.

**Visualization:** Jialong Liu, Chaojun Li.

**Writing – original draft:** Jialong Liu.

**Writing – review & editing:** Hanzhi Qin.

## Supplementary Material
















